# Inorganic Nitrogen-Containing Aerosol Deposition Caused “Excessive Photosynthesis” of Herbs, Resulting in Increased Nitrogen Demand

**DOI:** 10.3390/plants11172225

**Published:** 2022-08-27

**Authors:** Zhiwei Ge, Yunran Ma, Wei Xing, Yongbo Wu, Sili Peng, Lingfeng Mao, Zimei Miao

**Affiliations:** 1 College of Biology and the Environment, Co-Innovation Center for Sustainable Forestry in Southern China, Nanjing Forestry University, Nanjing 210037, China; 2 NFU Academy of Chinese Ecological Progress and Forestry Development Studies, Nanjing 210037, China; 3 Jiangsu Academy of Forestry, Nanjing 211100, China

**Keywords:** nitrogen contained aerosol, nitrogen use efficiency, superoxide dismutase activities, *Iris germanica* L., *Portulaca grandiflora* Hook

## Abstract

The amount of atmospheric nitrogen-containing aerosols has increased dramatically due to the globally rising levels of nitrogen from fertilization and atmospheric deposition. Although the balance of carbon and nitrogen in plants is a crucial component of physiological and biochemical indexes and plays a key role in adaptive regulation, our understanding of how nitrogen-containing aerosols affect this remains limited; in particular, regarding the associated mechanisms. Using a fumigation particle generator, we generated ammonium nitrate solution (in four concentrations of 0, 15, 30, 60 kg N hm^−2^ year^−1^) into droplets, in 90% of which the diameters were less than 2.5 μm, in the range of 0.35–4 μm, and fumigated *Iris germanica* L. and *Portulaca grandiflora* Hook. for 30 days in April and August. We found that the weight percentage of nitrogen in the upper epidermis, mesophyll tissue, and bulk of leaves decreased significantly with the N addition rate, which caused a decrease of carbon:nitrogen ratio, due to the enhanced net photosynthetic rate. Compared with *Portulaca grandiflora* Hook., *Iris germanica* L. responded more significantly to the disturbance of N addition, resulting in a decrease in the weight percentage of nitrogen in the roots, due to a lower nitrogen use efficiency. In addition, the superoxide dismutase activity of the two plants was inhibited with a higher concentration of nitrogen sol; a reduction of superoxide dismutase activity in plants means that the resistance of plants to various environmental stresses is reduced, and this decrease in superoxide dismutase activity may be related to ROS signaling. The results suggest that inorganic nitrogen-containing aerosols caused excessive stress to plants, especially for *Iris germanica* L.

## 1. Introduction

The extensive use of nitrogen-containing fertilizers, industrial emission, automobile exhaust, and fossil fuels has dramatically increased the amount of atmospheric nitrogen-containing aerosols [1]. Excessive deposition of nitrogen leads to soil acidification and water eutrophication, which threatens the stability of nitrogen-related ecological processes [2,3,4], and has a substantial negative impact on the structure and function of ecosystems globally [5,6,7,8]. According to their aerodynamic diameter, the aerosols in particulate matters (PM) can be classified as PM_10_ (Ø ≤ 10 μm), PM_2.5_ (Ø ≤ 2.5 μm), and PM_0.1_ (Ø ≤ 0.1 μm) [9]. Among them, PM_2.5_ is known for its difficult settlement, a wide range of influences, great harm to the human body, control difficulties due to a small particle size, and easy enrichment [10]. Furthermore, water soluble inorganic salt is the main component of PM_2.5_, and its contribution rate to the mass concentration of PM_2.5_ is more than 40% [11]. NH^4+^ and NO^3−^ are the main components of water-soluble inorganic salts [12]. Therefore, study of the environmental effects of inorganic-nitrogen-formed PM_2.5_ is important to understand the effect of aerosols on the physiological and ecological processes of animals and plants.

As a natural purifier to improve the environment, plants can effectively block and absorb aerosols and other particles in the air, and play a leading role in improving air quality [13,14]. The dust retention ability of plants is closely related to their leaf morphology and leaf surface characteristics. Leaves with a rough surface, or that are fluffy or mucus secreting, are more likely to absorb aerosols and other particles in the atmosphere [15]. Meanwhile, increased emissions of inorganic-nitrogen-formed aerosols have influences on plants, either via affecting the soil chemistry and other abiotic and biotic interactions, which have been well studied in the form of traditional nitrogen addition or fertilization [16,17,18], or via surface penetration on above ground organs directly [19], about which knowledge remains limited. It used to be believed that PM_0.1_, which represents only a small proportion of aerosols, was the main component that can pass through the plant stomata. However, Lehndorff et al. [20] showed evidence that PM_2.5_ can also penetrate into the leaves through the stomata, and it became the dominate component affecting physiological and biochemical process in leaves, due to its high proportion in aerosols comparing to PM_0.1_.

It has been reported that, due to the proportional dependence on carbon and nitrogen caused by the long-term evolution of plants, proper application of nitrogen to the leaves will lead to an increase of photosynthetic rate, to maintain the carbon and nitrogen balance [21,22], and some plants growing in adversity can also actively increase their photosynthetic rate, to improve nitrogen metabolism [23,24]. The high concentration of nitrogen in air can have a negative effect on the physiology and growth of individual plants [25,26], caused by the cellular acidosis and the destruction of electron transport in chloroplasts, which usually results in yellowing, slowed growth, and necrosis of leaves [27]. When a nitrogen-containing droplet enters the plant through the stomata on the leaves, it dissolves rapidly in the continuous area of the surrounding cell wall [28]. An overdose of nitrogen in the cell wall will inhibit the activity and content of photosynthetic enzymes in leaves, and eventually lead to a decrease of photosynthetic rate and carbon nitrogen ratios. At the same time, it can depress the production of NADP^+^ in chloroplasts, increase the content of active oxygen, and cause oxidative damage [29,30], which can be reflected by the change of superoxide dismutase (SOD) activity. SOD is the first antioxidant enzyme shown to play a role in the process of scavenging reactive oxygen species [31]. In addition, nitrous oxide dissolved in cells will be reduced to ammonium by nitrous reductase and then combined with other substances, to form macromolecular amino acids or proteins [32]. 

However, these results usually came from experiments spraying or smearing the nitrogenous solution on the leaves of fruit trees or other crops, while the spraying of nitrogen fertilizer on leaves, known as foliar fertilization, is an important management method in agriculture [33], in which the effect of nitrogen application is more through infiltration than through penetration into the sub-pores [34]. The nitrogen concentration in PM_2.5_ is usually represented by the unit μg/m^3^ [35,36] (such as 13.9–14.7 μg m^−3^ in 2004–2005 from Hangzhou City, China) [37], which is much less than the application concentration in foliar fertilization that usually uses the unit g N [38,39] (such as leaves spraying 0.5–1 g N plant^−1^ week^−1^ in a foliar fertilization experiment) [40]. In addition, the amount of nitrogen penetrating through stomata and participating in the process of metabolism and circulation in plants is also much less than the amount of nitrogen applied for fertilization. Therefore, whether the effects of inorganic nitrogen PM_2.5_ applications (with lower nitrogen concentrations and lesser penetration processes) on the photosynthesis and other physiological processes of plant leaves are all negative is worth exploring.

*Iris germanica* L. and *Portulaca grandiflora* Hook. are widely used garden plants and are representative C_3_ and C_4_ plants. The hypothesis that a C4 plant has much higher nitrogen use efficiency (NUE) than a C3 plant was put forward by Brown [5]. It can be summarized as follows: (1) C4 plants can assimilate NH_4_^+^ in both mesophyll cells and vascular bundle sheath cells, to synthesize amino acids and proteins, while C3 plants reduce nitrogen only in mesophyll cells, resulting in low NUE. (2) Compared with the CO_2_ fixed by C3, the nitrogen demand from the C4 photosynthetic pathway will increase correspondingly, to maintain the carbon–nitrogen balance [41]. Therefore, in the long-term evolution process, C4 evolved a complex biochemical adaptation mechanism to improve NUE, such as higher cytoplasmic nitratase [42,43], and lower Ribulose bisphosphate carboxylase/oxygenase (Rubisco) [44,45] and Calvin-Benson cycle enzyme contents [46]. Therefore, it has been reported that C4 plants suffer a competitive disadvantage during nitrogen addition treatments [47,48]. However, whether the amount of nitrogen penetrating across the stomata from PM_2.5_ is sufficient for the relief of nitrogen limitation remains unclear, although it had been reported that the photosynthetic rate of C3 plants increased slightly in the low concentration of gaseous nitrogen dioxide, without thorough discussion, because of the total decreased pattern, along with the increase of nitrogen addition concentration [49]. *Iris germanica* L. and *Portulaca grandiflora* Hook. have similar differences in light and coping, so experimental verification of their differences is required.

Therefore, inorganic nitrogen containing aerosol fumigation needs to be conducted to improve our understanding of the response of carbon and nitrogen balance in plants. The objectives of this study were (1) to examine the effects of inorganic nitrogen PM_2.5_ on photosynthetic rate and carbon/nitrogen assignment, and (2) to evaluate the different strategies of *Iris germanica* L. and *Portulaca grandiflora* Hook. under its influence, due to their different NUE.

## 2. Materials and Methods

### 2.1. Materials Preparation

*Iris germanica* L. and *Portulaca grandiflora* Hook. were chosen as the experiment materials, due to their common use as urban greening herb species. The two species were sown in a greenhouse in February with uniform nursery soil (Model 422, Klasmann-Deilmann GMBH Incorporated, Geeste, German), located in Jiangsu Academy of Forestry, Nanjing, China. Thirty two plants growing in unison for both species were chosen in April and August, which was the vegetative stage for both.

### 2.2. Experimental Design

**Figure 1:**
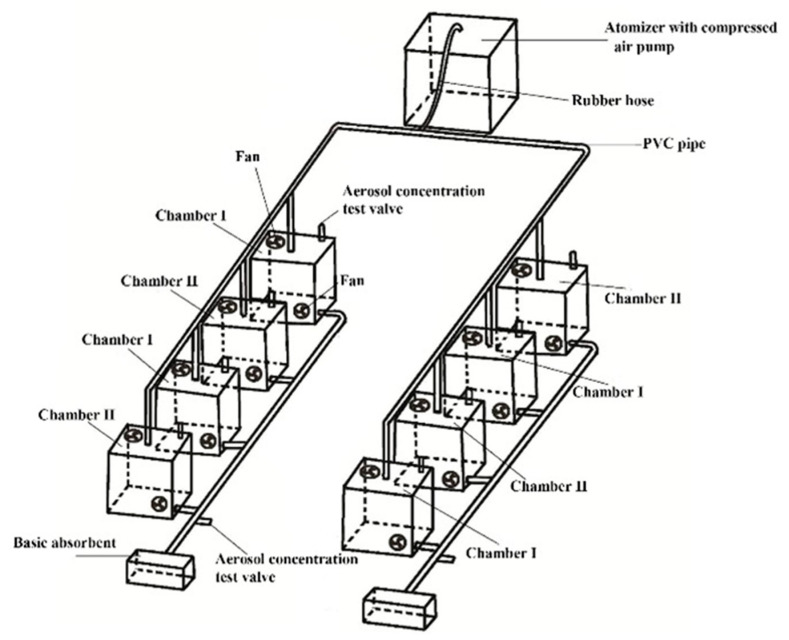
Experimental setup diagram of one application concentration of inorganic nitrogen fumigation. Chambers I and II were for the two types of tested plants (*Iris germanica* L. and *Portulaca grandiflora* Hook.). The aerosol concentration test valve was for the monitoring of aerosol concentration, which was only opened for concentration checking during fumigating. The basic absorbent was sodium bicarbonate solution. The fans were for the uniform distribution of aerosols, temperature, and CO_2_, vertically and horizontally.

A six-jet Atomizer (Model: 9306A, TSI Incorporated, Shoreview, MN 55126, USA) with compressed air pump (Model 36-7, Jiebao Incorporated, Shanghai, China) was used as the fumigation particle generator and generated ammonium nitrate solution into droplets, in 90% of which the diameters were less than 2.5 μm, in the range of 0.35–4 μm. This was calibrated by adjusting nozzle the valve and monitored using a Dusttrak II particulate monitor (Model: 8530, TSI Incorporated, Shoreview, MN 55126, USA). The plants were placed in a series of chambers, alternately connected with rubber hose and PVC pipe (Figure 1). The size of each chamber was 30 × 30 × 65 cm, with a side sliding door. In order to prevent nitrogen-contained aerosols from being absorbed by the soil, a plastic film was used to wrap the containers for planting plants, to eliminate the influence of fumigation on the soil. For watering, a PVC pipe with a lid on the top was inserted into the soil.

The concentration of aerosols was set at 50 μg/m^3^ through the adjustment of a flux valve, while the value of Nanjing city was 43–74 μg/m^3^ in 2014–2016 [50]. The nitrogen levels were 0, 15 (half dose), 30 (background dose), and 60 (double dose) kg N hm^−2^ year^−1^ (32 plants were needed for each species), while bulk deposition fluxes of inorganic nitrogen averaged 35.8 kg N hm^−2^ year^−1^, and wet deposition fluxes of inorganic nitrogen were 28.7 kg N hm^−2^ year^−1^ [51]. In order to maintain the uniformity of carbon dioxide and temperature, the control was fumigated using pure water vapor, to ensure the uniformity of environmental factors (such as temperature, CO_2_ concentration, etc.)

### 2.3. Sampling and Measurements

From 1 April to 30 April (non-growing season) and from 15 July to 15 August (growing season) 2018, under natural light conditions, leaves with the same status and complete shape were selected to measure the physiological indexes of net photosynthetic rate, transpiration rate, and stomatal conductance, using a portable photosynthetic instrument (Model: LI-6400, LI-COR Incorporated, Lincoln, Nebraska 68504, USA), which was completed before 12:00 a.m. in sunny weather (once each week during fumigations and lasting for one month (5 measurements)). On 1 May (non-growing season) and 16 August (growing season) of 2018, leaves with the same growth, complete shape, and healthy maturity from each plant were taken, and the roots were collected, from 10:00 a.m. to 12:00 a.m., and then stored with dry ice and brought back to the laboratory for testing. 

The preparation process was as follows: Weigh about 0.2 g of fresh leaves, cut them into pieces, add 10 mL of precooled PBS 7.8 solution (added twice), grind and extract, then centrifuge at 4 °C in 10,500 rpm for 15 min, take the supernatant, and store at 4 °C. SOD activity was tested with the nitro blue tetrazolium (NBT) photoreduction method [52]. An enzyme activity unit determined 50% inhibition of NBT photoreduction. It was calculated as follows:

SOD activity = [(ACK − AE) × V]/(ACK × 0.5 × W × Vt)(1)

where ACK is the light absorption value of illumination to the care; AE is the light absorption value of the sample tube; V is the total volume of sample solution; Vt is the dosage of sample solution in determination; and W is the weight of samples.

Total C and N were measured using an element analyzer (Elementar Vario EL, Hanau, Germany) from part of the leaves and roots, which had already been oven baked for 30 min at 105 °C, dried to constant weight at 55–65 °C, and ground. 

**Figure 2:**
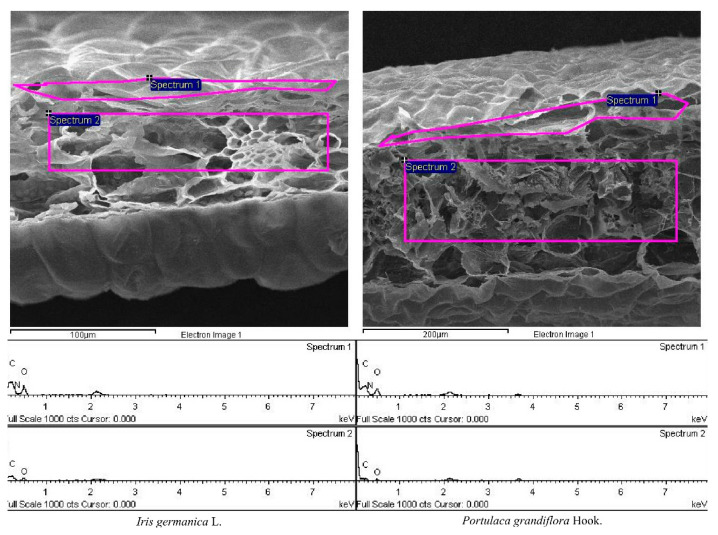
Example of field emission scanning electron microscope output for the leaves of two species. Spectrum 1 and 2 are the sample area for the weight percentage (%) of carbon (C), nitrogen (N), and oxygen (O) in the upper epidermis and mesophyll tissue of leaves, respectively.

The other parts of leaves were prepared as portrait section slices with a square blade, with side length of 5 mm, after freeze-drying, and the chemical composition of their upper epidermis and mesophyll tissue (Example diagram in Figure 2) was studied using a field emission scanning electron microscope (Model: JSM-7600F, JEOL Incorporated, Akishima, Japan). 

### 2.4. Statistical Analysis

To test the effects of species, nitrogen addition, weeks in fumigation, and season on the net photosynthetic rate, stomatal conductance, and the weight percentage of carbon and nitrogen in the upper epidermis, mesophyll tissue, and bulk of leaves, we used the following linear mixed model:

(2) $$Y_{ijklmn}=S_{n}+N_{i}+M_{j}+D_{k}+Interaction\;within\;S_{n},\;N_{i},\;M_{j}\;and\;D_{k}+\pi |B_{l}+\varepsilon_{m\left(ijkl\right)}$$

where *Y_ijklmn_* is the net photosynthetic rate, stomatal conductance, and the weight percentage of carbon and nitrogen in the upper epidermis, mesophyll tissue, and bulk of leaves; *S_n_* (*n* = 1, 2); *N_i_* (i = 0, 1, 2, 3) is the level of nitrogen application (0, 15, 30, 60 kg N hm^−2^ year^−1^); *M_j_* (*j* = 1, 2) is the month (April and August); *D*_k_ (*k* = 1, 2, 3, 4, 5) is sample date (3, 8, 15, 22, 30 days in fumigations), which was excluded in the analysis of the weight percentage of carbon and nitrogen, because they were measured only once at the end of each experimental period; *π*|B*_l_* represents the random plot effect (*l* = 1, 2, …16) nested in the two random blocks; and *ɛ_m(ijkl)_* (*m* = 1, 2) is the sampling error. We conducted a linear mixed effect analysis, using the restricted maximum likelihood estimation with the “*lme4*” package [53]. We then calculated the absolute values of spring and summer plant measurements: photosynthetic rate (Ph), transpiration rate (Tr), stomatal conductance (g_s_) and the corresponding standard errors (Table 1).

To better understand the mechanisms associated with changes in these physiological and biochemical indexes, we used Pearson correlation analysis, which was performed using the “PerformanceAnalysis” package [54], to examine the extent of associations. All analyses were performed using R Statistical Software (Version: 4.1.3, The R Foundation for Statistical Computing c/o Institute for Statistics and Mathematics, Vienna, Australia) [55]. 

## 3. Results

### 3.1. The Effect on Carbon and Nitrogen Distribution in Leaves

**Figure 3:**
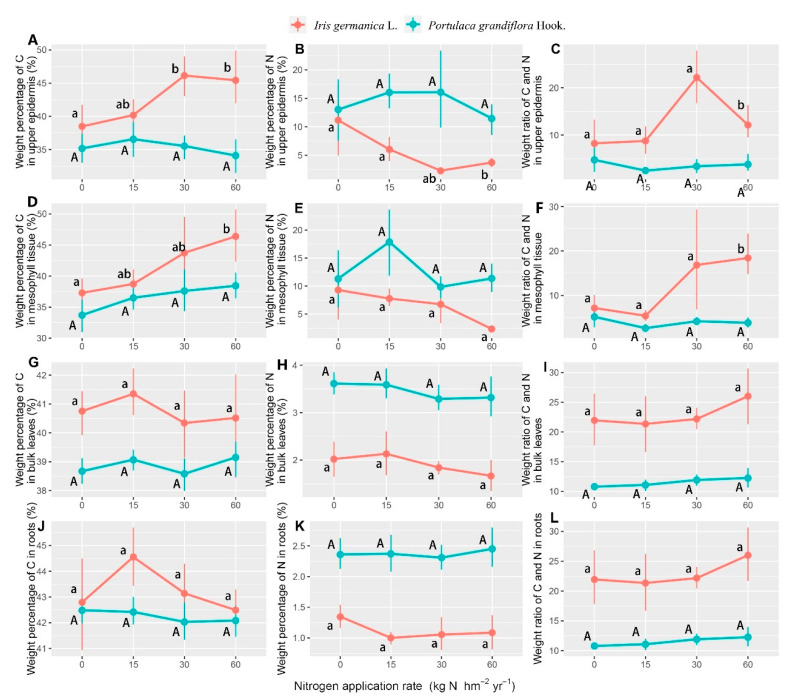
The response of the percentage weight of carbon (C), nitrogen (N), and C/N in the roots (R) (**A**–**C**), the upper epidermis (UE) (**D**–**F**), mesophyll tissue (MT) (**G**–**I**), and bulk of leaves (BL) (**J**–**L**) to the nitrogen application rate in the form of aerosols in *Iris germanica* L. and *Portulaca grandiflora* Hook. (averages of two sampling seasons). Values are means with 95% bootstrapped confidence intervals (CI). Differences are significant at α = 0.05, when the CI does not overlap the subsequent mean. Upper and lower case letters indicate the significantly different results of *Portulaca grandiflora* Hook. and *Iris Germanica* L. on applied nitrogen concentration, respectively.

Our result showed that the percentage weight of nitrogen was enhanced and carbon was depressed with the increase of nitrogen concentration in the form of aerosols, which caused an increase of carbon and nitrogen weight ratios. The concentration of inorganic nitrogen in the form of aerosols affected the nitrogen in the upper epidermis and the bulk of *Iris germanica* L. leaves, and only the percentage weight of carbon in the mesophyll tissue of its leaves varied significantly, while several interaction effects were statistically significant (Table 2). The weight ratios of carbon and nitrogen in the upper epidermis and mesophyll tissue of *Iris germanica* L. leaves increased significantly as the application was increased to 60 kg N hm^−2^ year^−1^, due to the dramatic increase (rising by 19.4% comparing to 0 kg N hm^−2^ year^−1^) of carbon and decrease of nitrogen (declined 26.4% comparing to 0 kg N hm^−2^ year^−1^), respectively, which was not found in *Portulaca grandiflora* Hook. (Figure 3A–F). Furthermore, the nitrogen containing PM_2.5_ induced a decrease of nitrogen percentage weight and caused a decrease of the nitrogen percentage weight in the bulk leaves, although it did not show significant differences for *Portulaca grandiflora* Hook., due to the considerable standard errors (Figure 3H).

### 3.2. Effect on the Net Photosynthetic Rate, Transpiration Rate, and Stomatal Conductance

**Figure 4:**
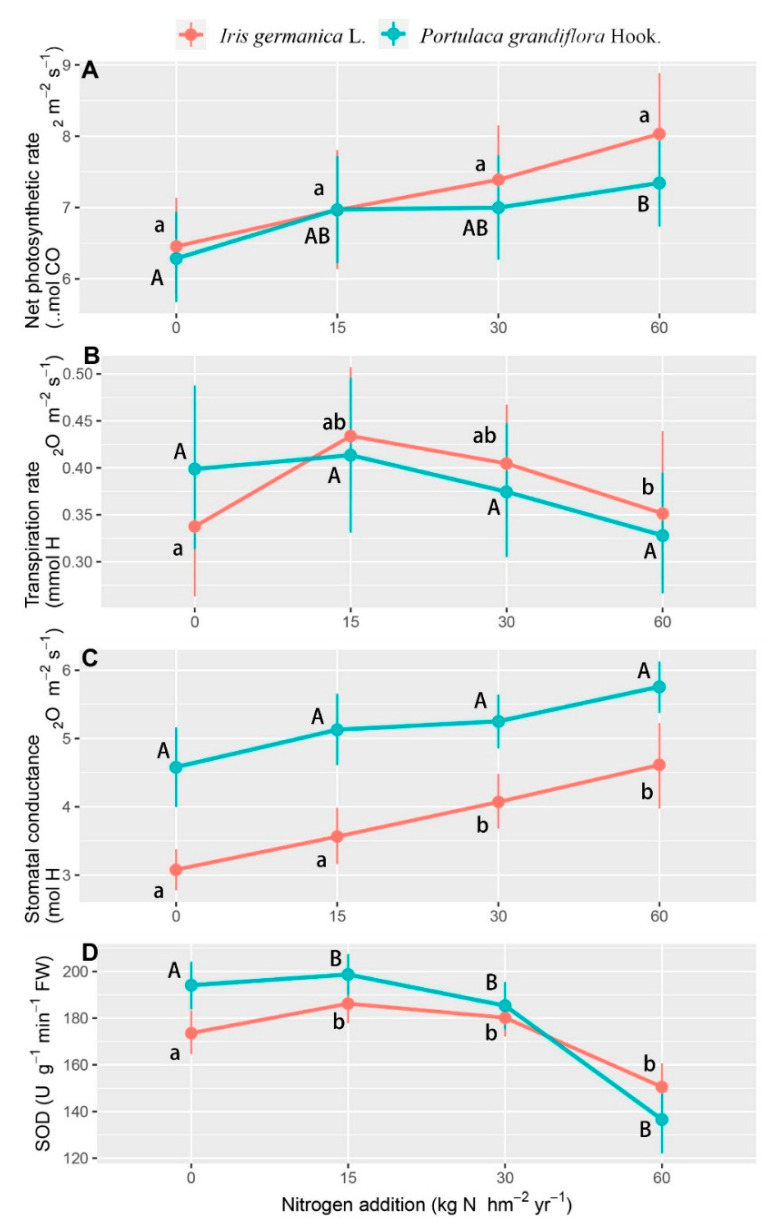
The response of the net photosynthetic rate (Npr) (**A**), transpiration rate (Tr) (**B**), stomatal conductance (g_s_) (**C**), and SOD activity (**D**) to nitrogen application rate in the form of aerosols in *Iris germanica* L. and *Portulaca grandiflora* Hook. over sampling dates. Values are means with 95% bootstrapped confidence intervals (CI). Differences are significant at α = 0.05, when the CI does not overlap the subsequent mean. Upper and lower case letters indicate significantly different results for *Portulaca grandiflora* Hook. and *Iris Germanica* L. on applied nitrogen concentration, respectively.

A significantly increased net photosynthetic rate and stomatal conductance were also found in response to the nitrogen application rate, while the transpiration rate and SOD decreased with the nitrogen application rate up to 30 and 60 kg N hm^−2^ year^−1^ (Table 3, Figure 4). The net photosynthetic rate showed no significant differences at the nitrogen application rates of 0 and 15 kg N hm^−2^ year^−1^, and *Iris germanica* L. showed a more significant increase up to the highest nitrogen application (Figure 4A), which was totally opposite for the SOD activities. *Portulaca grandiflora* Hook. had higher SOD activities at the nitrogen application rates of 0 and 15 kg N hm^−2^ year^−1^, but this effect disappeared under the trend of a rapid decline at higher nitrogen application rates (Figure 4D). The transpiration rate showed significant variation between the two species, but no significant variation existed for the stomatal conductance (Figure 4B,C).

### 3.3. Correlation Analysis between Possible Drivers and Nitrogen Distribution in Plants

**Figure 5:**
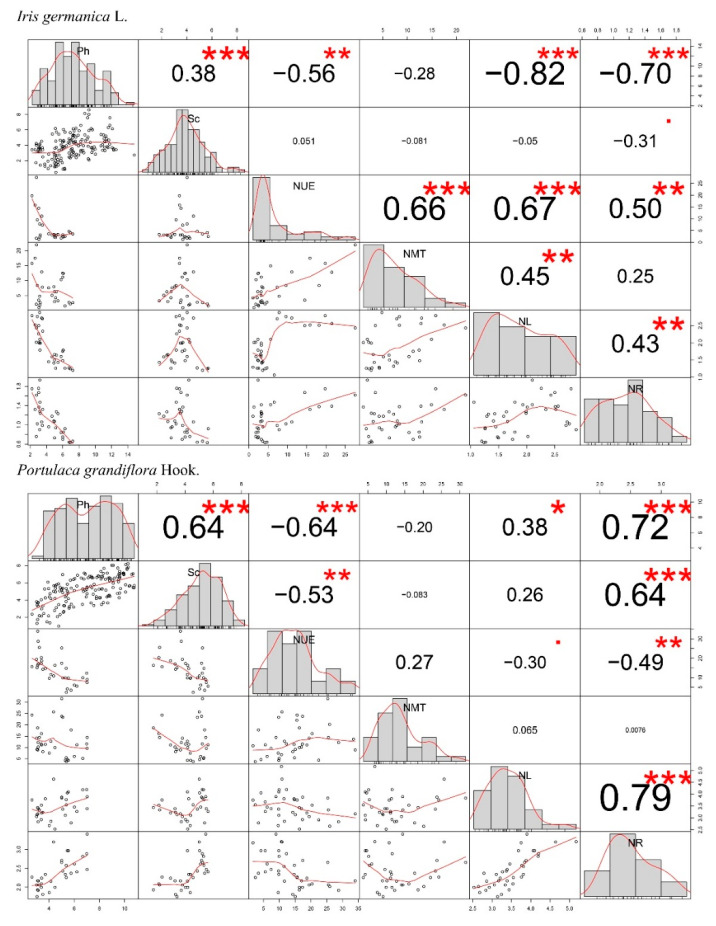
Pearson correlations between the net photosynthetic rate (Ph), stomatal conductance (g_s_), the weight percentage of nitrogen (N) in the upper epidermis (UE), mesophyll tissue (MT), bulk of leaves (BL), and in the roots (R). At the bottom of the plot, bivariate scatter plots with a smooth line are displayed. At the top of the plot, the value of the correlation plus the significance level are displayed as asterisks (*** *p* < 0.001, ** *p* < 0.01, * *p* < 0.05). Units associated with the variables are shown in Figure 3 and Figure 4.

According to the correlation analysis, the net photosynthetic rate played an important role in the decreasing weight percentage of nitrogen in the leaves of *Iris germanica* L. but did not show the same pattern for *Portulaca grandiflora* Hook. (Figure 5), which could explain the differences in Figure 3H between these two species. Although the correlation between the net photosynthetic rate and nitrogen weight percentage in the upper epidermis of leaves was still uniformly positive in the two species, the nitrogen densities in the leaves and roots showed a contrary pattern (negative), in which the main effects of nitrogen application concentration were also only significant in *Iris germanica* L. (Table 2). In addition, the Pearson correlation analysis also showed the positive relationship between (1) the net photosynthetic rate and the stomatal conductance, and (2) the nitrogen density of leaves and roots in both *Iris germanica* L. and *Portulaca grandiflora* Hook. (Figure 5).

## 4. Discussion

### 4.1. Nitrogen-Containing Aerosol Affected the Balance of Carbon and Nitrogen

It is common for plants to maintain their internal carbon and nitrogen balance by regulating net the photosynthetic rate, to a ensure normal physiological process, which was widely recognized as the theory of carbon nitrogen ratio and often used as the theoretical basis for the management of nitrogen fertilizer addition to promote photosynthesis [56,57]. However, our results show that a nitrogen-containing aerosol induced an excessive increase of net photosynthetic rate and led to an imbalance of carbon and nitrogen, showing that the percentage of nitrogen in plant leaves decreased. To our knowledge, this is the first report to prove that nitrogen enters leaves directly through a stomata-induced increase of carbon and nitrogen ratios, which could be explained by the formation of excess photosynthetic products.

The carbon and nitrogen assimilation process is closely related and interacts through competition for photosynthesis. The photoreaction of photosynthesis produces ATP and reduces ferredoxin as intermediates through photophosphorylation. The reduced ferritin temporarily fixes electrons by reducing NADP^+^ to NADPH (for CO_2_ assimilation), and nitrate and nitrite ions to ammonium ion (for the synthesis of nitrogen-containing substances), which both involve the coupling of carbon and nitrogen [58,59]. Therefore, exogenous nitrogen can significantly affect the photosynthetic rate of plants [60], even the formation of chloroplasts [61], because plants tend to maintain a stable carbon nitrogen ratio [62]. Our results also showed a trend of net photosynthetic rate increasing with the increase of nitrogen concentration (Figure 4A), but not an inhibition due to the overdose of nitrogen, as some research reported [63,64,65], which showed that the nitrogen applied in this study was still at a low concentration level for plants. 

However, a difference between this study and previous studies is the method of nitrogen application. Increases of photosynthetic rate caused by nitrogen application are mainly due to the active increase of stomatal conductance [66,67]. Nevertheless, in our case, the increase of stomatal conductance induced a further increase in the amount of nitrogen entering the pores and the sub-pores [68], which led to a temporary but large increase of nitrogen concentration in the gas exchange space in direct contact with the chloroplast. This is the mechanism by which “excessive photosynthesis” could happen. The chloroplast was misled by the increase of nitrogen concentration in the local tissues and formed excessive photosynthetic products, in order to balance the extra exogenous nitrogen. This can be supported by the aerosols dramatically inducing enhanced carbon nitrogen ratios in the upper epidermis and mesophyll tissue (Table 2). In addition, nitrogen can further closely regulate the activity of cell protective enzymes related to plant senescence, by regulating SOD activity, so as to regulate leaf senescence [69]. Our results showed that the decrease of relative nitrogen content (Figure 3) caused by excessive photosynthesis led to a decrease of SOD activity in leaves (Figure 4D), which may increase the malondialdehyde content in plants and induce the premature senescence of leaves [70].

### 4.2. The Different Response of Iris germanica L. and Portulaca grandiflora Hook. to Nitrogen Containing Aerosols

Our results showed that although the nitrogen application (*p* = 0.032) and species (*p* < 0.001) affected the percentage weight of nitrogen in leaves significantly, the response of *Iris germanica* L. (*p* = 0.043) and *Portulaca grandiflora* Hook. (*p* = 0.558) plants were different, which indicated that the depression of nitrogen percentage weight in leaves (Figure 3), due to “excessive photosynthesis”, had been alleviated in *Portulaca grandiflora* Hook. The results that the net photosynthetic rate of *Portulaca grandiflora* Hook. increased with the increase of nitrogen concentration, and that the change of leaf nitrogen mass percentage was different from that of *Iris germanica* L. (Figure 3 and Figure 4), may be explained by the following: The typical structural difference between C4 and C3 plants is the vascular bundle sheath cells with a Kranz ring structure [71]. C4 plants have a higher NUE, due to different characteristics of carbon metabolism (i.e., different positions of nitrogen reducing and assimilating enzymes in the cells) and different mechanisms of nitrogen absorption, reduction, and assimilation [5,72]. C4 plants can assimilate ammonium ions in both mesophyll cells and vascular bundle sheath cells, to synthesize amino acids and proteins, while C3 plants only reduce nitrogen in mesophyll cells, resulting in a low NUE and photosynthetic efficiency [73,74]. At the same time, in terms of cell anatomical structure, the distance between the veins of C4 plants is generally smaller than that of C3 plants, the density of veins is higher, and the number of mesophyll cell layers between veins of C4 plants is also less [75,76]. The higher NUE and photosynthetic efficiency of C4 plants caused a higher tolerance to the low nitrogen concentration, in the form of an aerosol-induced imbalance of carbon and nitrogen. This was also supported by the different correlation patterns between the net photosynthetic rate and weight percentage of nitrogen in leaves (Figure 5).

Furthermore, our results showed that the nitrogen in plant roots was transported upward, in order to alleviate the phenomenon of the decrease of nitrogen content in leaves, while the carbon and nitrogen balance in the leaves of *Iris germanica* L. was disturbed as discussed, which led to the significant decrease of nitrogen weight percentage in the roots (Figure 3K, Table 2). The organic nitrogen in the root system is transported to the leaves through the xylem, and glutamine and glutamate are formed through the assimilation reaction, to participate in nitrogen metabolism [77]. At the same time, the amino acids produced by nitrogen metabolism in the leaves can also be transported back to the root system through the phloem [78]. Therefore, there must be a linear positive correlation between the nitrogen content in leaves and roots [79,80], as shown in Figure 5. This determines that when the relative content of nitrogen in leaves decreases, in order to maintain the balance of carbon and nitrogen, the root system will increase the transport of nitrogen [81]. However, for *Iris germanica* L., with a low NUE, the additional nitrogen transport from roots could not offset the decline of nitrogen relative content caused by “excessive photosynthesis”, which eventually led to the decrease of nitrogen percentage weight in roots.

## 5. Conclusions

In summary, we found that the inorganic nitrogen-containing aerosols caused a decrease of nitrogen percentage weight in leaves, due to an excessively enhanced net photosynthetic rate, which led to an imbalance of carbon and nitrogen in the plants. *Portulaca grandiflora* Hook. had a higher tolerance due to its higher NUE, while the nitrogen weight percentage in the roots of *Iris germanica* L. was also affected. In addition, the SOD activities were depressed with higher nitrogen concentrations of aerosols for both species, which might have caused the premature senescence of leaves; hence, the effect of inorganic nitrogen containing aerosols on plants was generally negative.

## Figures and Tables

**Table 1:** The absolute values of plant measurements in spring and summer: photosynthetic rate (Ph), transpiration rate (Tr), stomatal conductance (g_s_), and corresponding standard errors.

Level of Nitrogen Application (kg N hm^−2^ year^−1^)	0			15			30			60		
*Iris germanica* L.	Ph	Tr	g_s_	Ph	Tr	g_s_	Ph	Tr	g_s_	Ph	Tr	g_s_
April	7.03	0.32	3.02	7.47	0.42	3.24	7.78	0.29	4.69	8.18	0.26	6.19
Standard error	±0.38	±0.05	±0.22	±0.53	±0.05	±0.22	±0.48	±0.03	±0.22	±0.49	±0.03	±0.28
August	5.88	0.36	3.14	6.46	0.45	3.88	7.00	0.51	3.45	7.88	0.45	3.04
Standard error	±0.60	±0.05	±0.22	±0.69	±0.06	±0.35	±0.65	±0.04	±0.30	±0.79	±0.07	±0.29
*Portulaca grandiflora* Hook.												
April	6.81	0.33	6.19	7.28	0.35	6.32	7.22	0.30	6.13	7.36	0.29	6.46
Standard error	±0.41	±0.05	±0.24	±0.52	±0.05	±0.25	±0.45	±0.04	±0.16	±0.42	±0.05	±0.2
August	5.76	0.47	2.97	6.66	0.48	3.93	6.77	0.45	4.37	7.33	0.37	5.06
Standard error	±0.51	±0.07	±0.26	±0.61	±0.07	±0.26	±0.57	±0.06	±0.24	±0.53	±0.05	±0.30

**Table 2:** The effects of nitrogen-containing PM_2.5_ addition concentration (N), sampling date (M), and their interaction (N × M) on the weight percentage of carbon (C), nitrogen (N), and C/N in the upper epidermis (UE), mesophyll tissue (MT), bulk of leaves (BL), and roots (R) in *Iris germanica* L. and *Portulaca grandiflora* Hook. The linear mixed-effects model used the Kenward–Roger method as a denominator of degrees of freedom.

Source	C in UE			N in UE			C in MT			N in MT			C in BL			N in BL		
*Iris germanica* L.	SS	F	*p*	SS	F	*p*	SS	F	*p*	SS	F	*p*	SS	F	*p*	SS	F	*p*
N	405.9	4.0	0.105	**354.2**	**7.9**	**0.036**	**472.7**	**8.0**	**0.034**	89.3	2.1	0.258	4.7	0.3	0.846	**1.0**	**7.1**	**0.043**
M	9.5	0.4	0.548	**520.0**	**47.1**	**<0.001**	**454.9**	**30.8**	**<0.001**	28.2	2.7	0.116	0.2	0.1	0.818	**8.0**	**225.5**	**<0.001**
N × M	174.1	1.7	0.181	**310.3**	**9.4**	**<0.001**	**331.6**	**5.6**	**0.002**	**438.1**	**13.7**	**<0.001**	6.5	0.5	0.762	**0.9**	**6.3**	**0.001**
*Portulaca grandiflora* Hook.																		
N	51.8	0.9	0.539	144.4	1.4	0.387	141.3	2.0	0.265	311.3	4.9	0.072	4.4	1.7	0.306	0.7	0.9	0.558
M	5.2	0.4	0.553	**1045.4**	**39.4**	**<0.001**	7.3	0.4	0.530	12.2	0.8	0.387	**6.9**	**10.6**	**0.003**	**4.4**	**20.9**	**<0.001**
N × M	39.3	0.7	0.611	**503.9**	**4.7**	**0.005**	79.2	1.1	0.379	**1026.0**	**16.4**	**<0.001**	1.4	0.6	0.699	1.0	1.2	0.318
**Source**	**C in R**			**N in R**			**C/N in UE**			**C/N in MT**			**C/N in BL**			**C/N in R**		
*Iris germanica* L.	SS	F	*p*	SS	F	*p*	SS	F	*p*	SS	F	*p*	SS	F	*p*	SS	F	*p*
N	22.53	1.92	0.271	**1.01**	**5.94**	**0.056**	**1071.51**	**4.81**	**0.080**	**1069.88**	**6.65**	**0.052**	112.14	3.43	0.132	**1789.5**	**7.28**	**0.040**
M	**31.01**	**10.58**	**0.003**	**1.41**	**33.16**	**<0.001**	**436.02**	**7.97**	**0.010**	**135.04**	**3.44**	**0.077**	**1166.79**	**142.67**	**<0.001**	**2136.6**	**34.78**	**<0.001**
N × M	20.63	1.76	0.169	**0.79**	**4.65**	**0.006**	**588.47**	**3.58**	**0.032**	**437.44**	**11.08**	**<0.001**	**118.09**	**3.61**	**0.019**	**1215.2**	**4.95**	**0.004**
*Portulaca grandiflora* Hook.																		
N	1.28	0.89	0.543	0.23	0.74	0.612	36.09	0.60	0.681	24.82	2.59	0.185	11.45	1.52	0.345	10.0	0.67	0.648
M	**8.89**	**24.73**	**<0.001**	**4.22**	**54.53**	**<0.001**	**143.14**	**9.58**	**0.005**	**12.89**	**5.39**	**0.029**	**56.77**	**30.18**	**<0.001**	**186.45**	**49.82**	**<0.001**
N × M	**5.33**	**3.71**	**0.018**	0.12	0.39	0.814	62.85	1.05	0.401	**97.35**	**10.18**	**<0.001**	14.03	1.86	0.151	7.25	0.48	0.747

**Note**: Bold font indicates statistical significance (α = 0.10). The columns provide the sum squares (SS), F and *p* values.

**Table 3:** The effects of inorganic nitrogen PM_2.5_ (N), species(S), days in fumigation (D), experimental month (M), and their interactions on the net photosynthetic rate, transpiration rate, and stomatal conductance of *Iris germanica* L. and *Portulaca grandiflora* Hook. The linear mixed-effects model used the Kenward–Roger method as a denominator for degrees of freedom.

Source	Net Photosynthetic Rate			Transpiration Rate			Stomatal Conductance				SOD	
	SS	F	*p*	SS	F	*p*	SS	F	*p*	SS	F	*p*
N	**71.47**	**79.55**	**<0.001**	**0.30**	**7.67**	**0.013**	**77.48**	**242.39**	**<0.001**	**229,090**	**83.45**	**<0.001**
S	**7.71**	**25.73**	**0.001**	0.0008	0.06	0.812	**145.46**	**1365.07**	**<0.001**	**5821**	**6.36**	**0.040**
D	**1503.44**	**1255.02**	**<0.001**	**8.28**	**158.03**	**<0.001**	**275.77**	**647.00**	**<0.001**	**277,801**	**75.90**	**<0.001**
M	**36.18**	**120.79**	**<0.001**	**1.21**	**92.28**	**<0.001**	**191.58**	**1797.92**	**<0.001**	**9778**	**10.69**	**0.001**
N × S	**5.37**	**5.98**	**0.024**	0.11	2.84	0.115	**2.79**	**8.73**	**0.009**	**26,032**	**9.48**	**0.007**
N × D	**1961**	**5.45**	**<0.001**	**0.44**	**2.83**	**0.001**	**7.39**	**5.78**	**<0.001**	**40,377**	**3.68**	**<0.001**
S × D	**25.04**	**20.90**	**<0.001**	**0.96**	**18.40**	**<0.001**	**47.52**	**111.49**	**<0.001**	**115,819**	**31.64**	**<0.001**
N × M	**9.21**	**10.25**	**<0.001**	**0.14**	**3.51**	**0.016**	**19.73**	**61.73**	**<0.001**	**15,244**	**5.55**	**<0.001**
S × M	**1.46**	**4.88**	**0.028**	0.0003	0.02	0.878	**32.87**	**308.45**	**<0.001**	**109,988**	**120.20**	**<0.001**
D × M	**168.46**	**140.62**	**<0.001**	**1.43**	**27.38**	**<0.001**	**25.43**	**59.67**	**<0.001**	**112,926**	**30.85**	**<0.001**
N × S × D	**6.86**	**1.91**	**0.034**	**0.62**	**3.94**	**<0.001**	**3.36**	**2.63**	**<0.001**	**37,932**	**3.45**	**<0.001**
N × S × M	0.22	0.25	0.861	**0.18**	**4.60**	**0.004**	**85.57**	**267.68**	**<0.001**	1905	0.69	0.556
N × D × M	**29.89**	**8.32**	**<0.001**	**0.91**	**5.79**	**<0.001**	1.79	1.40	0.167	**37,940**	**3.46**	**<0.001**
S × D × M	1.89	1.58	0.180	0.05	1.02	0.400	**2.03**	**4.75**	**0.001**	**60,407**	**16.50**	**<0.001**
N × S × D × M	**9.87**	**2.75**	**0.002**	**0.66**	**4.19**	**<0.001**	**5.20**	**4.06**	**<0.001**	19,172	1.75	0.054

**Note**: Bold font indicates statistical significance (α = 0.05). The columns provide the sum squares (SS), F and *p* values.

## Data Availability

Not applicable.
